# Mathematical modeling and analysis of spinal circuits involved in locomotor pattern generation and frequency-dependent left-right coordination

**DOI:** 10.1186/1471-2202-15-S1-P53

**Published:** 2014-07-21

**Authors:** Yaroslav I Molkov, Bartholomew J Bacak, Ilya A Rybak

**Affiliations:** 1 Department of Mathematical Sciences, Indiana University – Purdue University Indianapolis, IN 46202, USA; 2 Department of Neurobiology and Anatomy, Drexel University College of Medicine, Philadelphia, PA 19129, USA

## 

Coordination between left and right neural activities in the spinal cord during locomotion is controlled by commissural interneurons (CINs). Several CIN types have been genetically identified, including the excitatory V3 and excitatory and inhibitory V0 types. Talpalar et al. [1] recently reported that genetic elimination of the V0 CINs caused switching from a normal left-right alternating pattern of motor activity to a left-right synchronized “hopping” pattern. Furthermore, ablation of only the inhibitory V0 neurons (V0_D_ subtype) resulted in a lack of left-right alternation at low locomotor frequencies and maintaining this alternation at high frequencies, whereas selective ablation of the excitatory V0 neurons (V0_V_ subtype) maintained the left–right alternation at low locomotor frequencies and switched the motor output to a left-right synchronized ("hopping") pattern at high frequencies.

**Figure 1:**
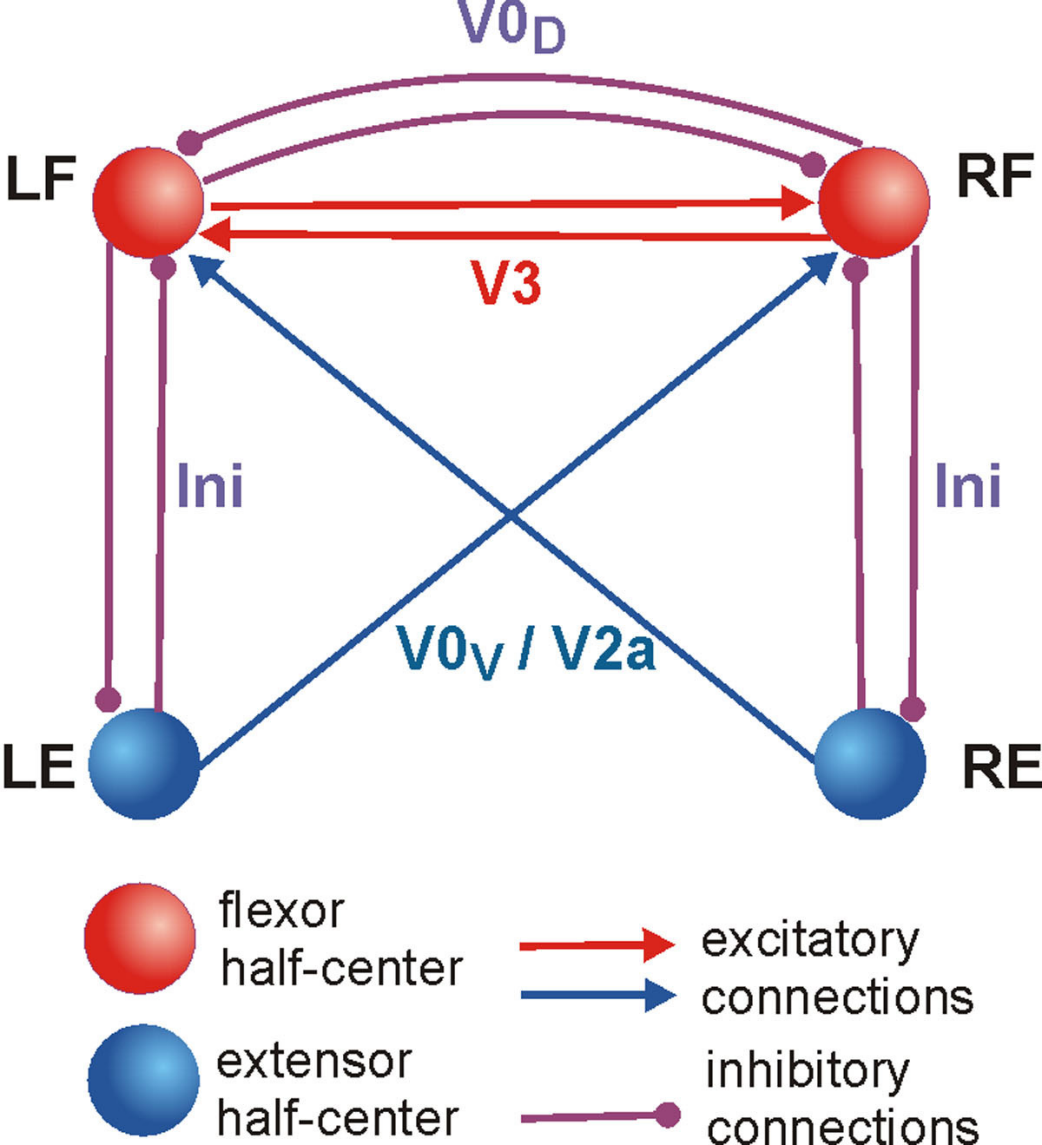
Model schematic.

To analyze and explain the above findings, we developed a simplified mathematical model of neural circuits consisted of four pacemaker neurons representing left (LF) and right (RF) flexor and left (LE) and right (RE) extensor half-centers interacting via commissural pathways representing V3, V0_D_, and V0_V_ CINs (Fig. 1). The “locomotor” frequency in the model was controlled by a parameter defining the excitability of neurons (via the leak reversal potentials) and commissural pathways, whose changes represented the corresponding changes induced by changing the concentration of N-methyl-D-aspartate (NMDA) applied to control the locomotor frequency in the isolated rodent spinal cord preparations [1].

The model demonstrated: (1) a typical left-right alternating pattern under control conditions; (2) switching to a synchronized hopping activity at any frequency after removing commissural connections representing both V0 (V0_D_ and V0_V_) neurons; (3) a synchronized left-right pattern at low frequencies and left-right alternation of activity at high frequencies after removing the commissural connections representing V0_D_ neurons; (4) an alternating left-right pattern at low frequencies with synchronized hopping at high frequencies after removing the commissural connections representing V0_V_ neurons. We used the bifurcation and fast–slow decomposition methods to analyze the behavior of this network in the four above states/regimes and transitions between them. The model was able to reproduce and provide explanation to several important experimental phenomena and generated predictions that can be tested experimentally.
